# Psychometric evaluation of the subjective well-being measure GP-CORE in a group of older adults in Sweden

**DOI:** 10.1186/s12877-022-03625-z

**Published:** 2022-11-28

**Authors:** Jacek Hochwälder, Lena-Karin Gustafsson, Gunnel Östlund, Viktoria Zander, Magnus L. Elfström

**Affiliations:** 1 Division of Psychology, School of Health, Care and Social Welfare, Mälardalen University, Eskilstuna, Sweden; 2 Division of Caring Science, School of Health, Care and Social Welfare, Mälardalen University, Eskilstuna, Sweden; 3 Division of Social work, School of Health, Care and Social Welfare, Mälardalen University, Eskilstuna, Sweden; 4 Division of Health and Welfare Technology, School of Health, Care and Social Welfare, Mälardalen University, Eskilstuna, Sweden; 5 Division of Psychology, School of Health, Care and Social Welfare, Mälardalen University, Eskilstuna, Sweden

**Keywords:** Geriatric mental health, GP-CORE, Older adults, Psychometrics, Subjective well-being

## Abstract

**Background:**

The world’s growing population of older adults is one population that needs to be focused more regarding subjective well-being. It is therefore important to evaluate self-report instruments that measures general well-being for this specific group - older adults. The aim of the present study was to investigate psychometric properties of the Swedish translation of the GP-CORE (general population – Clinical Outcomes in Routine Evaluation) in a group of older adults (> 65 years).

**Methods:**

In this study, a psychometric evaluation of the GP-CORE is presented for 247 Swedish older adults (> 65 years), 184 women and 63 men who applied for home care assistance for the first time.

**Results:**

The psychometric evaluation showed high acceptability; provided norm values in terms of means, standard deviations and quartiles; showed satisfactory reliability in terms of both internal consistency and stability; showed satisfactory validity in terms of convergent and discriminant validity; provided a very preliminary cut-off value and quite low sensibility and sensitivity and showed results which indicated that this scale is sensitive to changes. One gender difference was identified in that women without a cohabitant had a higher well-being than men without a cohabitant (as measured by GP-CORE).

**Conclusions:**

The GP-CORE showed satisfactory psychometric properties to be used to measure and monitor subjective well-being in older adults (> 65 years) in the general population of community dwelling. Future studies should establish a cut-off value in relation to another well-being measure relevant for mental health in older adults.

## Background

One population that needs to be focused more regarding subjective well-being is the world’s growing population of older adults. Indeed, the WHO based the Mental health action plan 2013–2020 on a life-course approach, underlining the need of well-being for older adults [1]. Moreover, it is necessary to recognise the gender differences in well-being that exist related to older age, especially recognisable in social and dyadic assets [2]. In Sweden, which is the empirical context of present study, women in average face five more years than men [3]. These years are often lived in single households without the caring responsibilities the women had during the previous part of the life span [4].

However, validated well-being measures for older adults are few, and recently a validation of one of the most well-known eudaimonic well-being questionnaires (i.e., the Scales of Psychological Well-Being by Ryff, [5]) called for further methodological consideration to optimize the care assessment of persons in old age [6]. Validated instruments that include older adults’ subjective wellbeing can further be useful in developing evidence-based knowledge on active aging [7] rather than to merely identify psychiatric needs. There are clear empirical indications that well-being can promote the overall health throughout an individual’s life e.g. [8–10]. In definitions of mental health, at least two aspects of subjective well-being tend to be presented [9, 11] i.e., a hedonic aspect, including emotional well-being and life satisfaction, as well as a eudemonic aspect including psychological and social well-being. In line with this way of thinking, the Clinical Outcomes in Routine Evaluation Outcome Measure (CORE-OM) was developed to meet the need of a self-report instrument that measures general well-being [12]. With its 34 items, the CORE-OM has indicated good psychometric properties [13, 14].

From CORE-OM a shorter scale was extracted, the General population – Clinical Outcomes in Routine Evaluation measure (GP-CORE) [15]. Sinclair et al. [15] decided that the item reduction should be driven by acceptability to users (relevant for people in general and consequently meaningful to answer), and that positive keyed and low intensity items should be retained, whereas all items connotating risk of self-harm or aggression towards others were deleted. Following the definition of subjective well-being [6, 8], the GP-CORE mirrors hedonic well-being by the conceptual domains well-being and problems/symptoms, whereas eudaimonic well-being is mirrored in the domain life/social functioning. In line with the CORE literature, we recommend that the emphasis in both research and clinical practice should be on the mean item score for all items, with domain scores only explored where particularly indicated clinically or for specific research interest [16].

Sinclair et al. [15] evaluated the GP-CORE regarding its psychometric properties in a group of student samples and found it had good psychometric properties in terms of reliability (internal-consistency and test-retest stability), convergent validity, detection of differences between clinical and non-clinical groups, and sensitivity to change [17]. However, for an instrument to be of value, it must have been evaluated on the population for which it is intended to be used [15, 18, 19]. The CORE-OM has been evaluated for a sample of older adults (> 65 years) from UK [12] but GP-CORE has, to our knowledge, not been evaluated for older adults. The CORE-OM has been translated to Swedish and psychometrically evaluated for a student sample and for a clinical sample [16] but a Swedish version of GP-CORE has not been evaluated. Thus, the aim of the present study was to investigate psychometric properties of the Swedish translation of the GP-CORE in a group of older adults (> 65 years).

## Methods

### Procedure

All older adults over 65 years applying to receive homecare for the first time delivered by the municipality in a middle-sized Swedish municipality (100,000 inhabitants) were asked to participate. The exclusion criteria were severe cognitive dysfunction (diagnosed), life-threating disease (e.g., a serious form of cancer), severe psychiatric diagnosis, or another disease or other barriers that disabled the person from expressing needs. The identified participants were randomized to an intervention group or to a control group that is described elsewhere (ClinicalTrials.gov NCT03565614). A power analysis based on quality of lite change in the pilot study of the intervention, and an estimated drop-out rate of 20%, showed that at least 240 persons in total should be recruited to the larger trial. The intervention group received intensive short-term reablement for three months. The general aim of the intervention was to maintain or increase the participants’ physical, psychological, and social functional abilities, whereas the control group received traditional homecare.

The data for this study was collected by a questionnaire, answered independently by each participant with support of the care managers, trained by the research team to explain and give information. The questionnaire was administered in personal meetings at three points in time: the first data collection was at the baseline or inclusion (Time 1); the second was post intervention three months after the baseline (Time 2); and the final and third was follow-up six months after the baseline (Time 3). Because there were no significant differences between the intervention group and the control group at any of the three measurement occasions regarding the GP-CORE, the two groups were combined into one group.

### Ethical approval

All persons were informed that their participation was voluntary and that all collected data would be handled confidentially. This study was approved by the Regional Ethic Review Board, `blinded for anonymity´ and met the ethical requirements consistent with the Helsinki declaration related to human research [20].

### Participants

330 persons that met the inclusion criteria were invited to participate, of which 247 persons (RR=74.8%; M_Age_=83.44 yrs; SD_Age_=7.39 yrs).184 women and 63 men, accepted to participate at Time 1. Demographic background variables at Time 1 are presented in Table 1. At Time 2, 227 persons participated (RR=91.9%; M_Age_=83.52, SD_Age_=7.40 yrs), 174 women and 53 men. At Time 3, 202 persons participated (RR=88.8%; M_Age_=83.44, SD_Age_=7.48 yrs), 157 women and 45 men.

**Table 1:** Description of the respondents of the present study on the demographic variables at Time 1

Variables	Total sample (*n* = 247)	Women (*n* = 184)	Men (*n* = 63)
Age			
M (SD)	83.44 (7.39)	83.90 (7.28)	82.11 (7.58)
Min-Max	65–99	66–99	65–97
Education			
Elementary school	52.6% (130)	53.8% (99)	49.2% (31)
Gymnasium or University	47.4 (117)	46.2% (85)	50.8% (63)
Cohabitant			
Yes	27.9% (69)	19.0% (35)	54.0% (34)
No	72.1% (178)	81% (149)	46.0% (29)
Diagnostic illnesses			
Yes	90.7% (224)	90.2% (166)	92.1% (58)
No	9.3% (23)	9.8% (18)	7.9% (5)
Medicate regularly			
Yes	99.6% (246)	99.5% (183)	100% (63)
No	0.4% (1)	0.5% (1)	0% (0)

### Questionnaire

Background variables were gender (man, woman); age (in years); education (elementary school/gymnasium, university); cohabitant (yes, no); diagnosed illness (yes, no); and regularly medicate (yes, no). EQ-5D-5L was developed by the EuroQoL Group to measure health-related quality of life and consists of six items (see for example [21–23]. The first five items are: mobility (walking about), self-care (washing and dressing), usual activities (e.g., work, study, and housework), pain/discomfort, and anxiety/depression. For each of these five items the respondents are asked to rate themselves with reference to how they feel today and the ratings for each item are given on a 5-point scale, where 1 = `no problems`, 2 = `slight problems`, 3 = `moderate problems`, 4 = `severe problems`, 5 = `extreme problems` (in walking about, with washing or dressing, in doing usual activities, with pain or discomfort, and with anxiety or depression). The sixth and final item is about general health. The ratings are given on a vertical visual analogue scale ranging from 0 (=`the worst health you can imagine`) to 100 (=`the best health you can imagine`). The six variables were treated separately in this study. General population – Clinical Outcomes in Routine Evaluation measure (GP-CORE) was developed by Sinclair et al. [15] and translated to Swedish by Elfström et al. [16] (Table 2). For each item the respondents rate themselves with reference to the past week. The ratings are given on a 5-point scale, where: 0 = not at all, 1 = only occasionally, 2 = sometimes, 3 = often, 4 = most or all of the time. As can be seen in Table 2, responses to eight of the fourteen items must be reversed before a single overall score is created by calculating arithmetic mean of the ratings to the 14 items, where higher values on this index indicates lower degrees of well-being [15].

**Table 2:** Description of GP-CORE

Item ^a^	English original	Swedish translation	Wording	Intensity	Domain
1.	I have felt tense, anxious or nervous	Jag har känt mig spänd, ängslig eller nervös	Negative	Low	Problems-anxiety
2. ^b^	I have felt I have someone to turn to when things go wrong	Jag har känt att jag haft någon att vända mig till när det behövts	Positive	Low	Functioning-close
3. ^b^	I have felt OK about myself	Jag har känt mig nöjd med mig själv	Positive	Low	Subjective well-being
4. ^b^	I have felt able to cope when things go wrong	Jag har känt att jag kunnat klara situationer där något gått snett	Positive	High	Functioning-general
5.	I have been troubled by aches, pains or other physical symptoms	Jag har besvärats av. värk, smärta eller andra kroppsliga problem	Negative	Low	Problems-physical
6. ^b^	I have been happy with the things I have done	Jag har varit nöjd med de saker som jag har gjort	Positive	Low	Functioning-general
7.	I have had difficulty getting to sleep or staying asleep	Jag har haft svårigheter att somna in eller att sova en hel natt	Negative	Low	Problems-physical

*Note*. ^a^ The answers are given on a 5-point Likert-scale (with reference to the past week), where: 0 = “not at all”, 1 =  “only occasionally”, 2 = “sometimes”, 3 = “often”, 4 = “most or all of the time”. ^b^ Responses to these items must be *reversed*

### Analyses

Most analyses were performed for data at Time 1 (baseline) because the number of participants was greatest at Time 1. The following statistical descriptions and analyses were performed: (1) Acceptability was estimated at Time 1 by computing the number (and percent) of participants that had answered all 14 questions of GP-CORE and the number (and percent) of persons that had not responded to one or several questions of GP-CORE. (2) Univariate statistical description of GP-CORE was made at Time 1 by computing arithmetic means, standard deviations, min-max values, percentiles, skewness statistics, and kurtosis statistics. (3) Reliability was evaluated in form of internal consistency by computing Cronbach’s alpha coefficients at Time 1 and in form of test-retest stability by computing intra-class correlation coefficient (ICC) – mixed model, absolute type, average measures - for the test-retest group. (4) Convergent and discriminant validity was evaluated at Time 1 by computing correlations (Spearman’s) between GP-CORE and the four background measures and the six health-related quality of life questions from EQ-5D-5L. (5) A cut-off value for GP-CORE at Time 1 was computed based on a median split of scores on the general health rating in EQ-5D-5L [for those that had rated a bad health (0–49 points on the self-rated health scale in EQ-5D-5L) and for those that had rated good health (50–100 points on the self-rated health scale in EQ-5D-5L)] and using the calculated cut-off value sensitivity and specificity was estimated. (6) Finally, sensitivity for change was evaluated - using the longitudinal data (Time 1, 2, and 3) - by performing a 2 (Gender) × 3 (Time) ANCOVA, with cohabitant as the covariate and GP-CORE as the dependent variable and also by computing the number (and percent) of participant with reliable improvement, reliable deterioration, and no reliable change between Time 1 and Time 2, between Time 1 and Time 3, and between Time 2 and Time 3.

## Results

### Acceptability

Of the 247 participants at Time 1 (baseline), 87.9% (217 persons) answered all 14 questions of the GP-CORE, 8.5% (21 persons) did not responded to one question, 2.8% (7 persons) to two questions, 0.4% (1 person) to four questions and 0.4% (1 person) to five questions. The non-response rates for the fourteen questions are as follows: Q1 (0.4%, 1 person); Q2 (0.8%, 2 persons); Q3 (1.6%, 4 persons); Q4 (5.7%, 14 persons); Q5 (0%, 0 persons); Q6 (1.2%, 3 persons); Q7 (0%, 0 persons); Q8 (1.2%, 3 subjects); Q9 (0.4%, 1 subject); Q10 (0.4%, 1 subject); Q11 (0%, 0 subjects); Q12 (1.2%, 3 persons); Q13 (2.4%, 6 persons); Q14 (2.4%, 6 persons). All participants answered the following; “I have been troubled by aches, pains or other physical symptoms.` (Q5), “I have had difficulty getting to sleep or staying asleep.` (Q7), and “I have felt unhappy.` (Q11).

### A univariate statistical description

Table 3 shows that for the main group, the average value on GP-CORE was 1.36, with a standard deviation of 0.61. The differences between men and women were not significant (*t*_245_ = − 0.98, *p* > .05, Cohen’s *d* = 0.12). The positive values for the skewness statistic implies that the distributions are positively skewed but the skewness statistic divided by its *SE* was less than two which indicates that the scale did not significantly deviate from a symmetric distribution (see SPSS) [24, 25]. Similarly, the kurtosis statistic implies that the distributions deviated from normality, but the kurtosis statistic divided by its *SE* was greater than two only for women, which indicates that the scale was significantly platykurtic only for women (see SPSS) [24, 25].

**Table 3:** Statistical measures for GP-CORE at Time 1

Measures	Total sample (n = 247)	Women (n = 184)	Men (n = 63)
M (95%-Confidence interval)	1.36 (1.29–1.44)	1.38 (1.30–1.48)	1.30 (1.16–1.44)
SD	0.61	0.62	0.66
Min	0	0	0.29
Max	3.14	3.14	2.57
25th percentile	0.92	0.92	0.92
50th percentile	1.29	1.30	1.21
75th percentile	1.79	1.86	1.64
Skewness (SE)	0.20 (0.16)	0.15 (0.18)	0.33 (0.30)
Kurtosis (SE)	−0.55 (0.31)	−0.56* (0.18)	−0.48 (0.60)

***p < .001, **p < .01, *p < .05

## Reliability

### Internal consistency

The Cronbach alpha coefficient for the total group was 0.79 (95% *CI*: 0.74 to 0.83). For the women, the alpha was 0.80 (95% *CI*: 0.75 to 0.84). For the men, the alpha was 0.74 (95% *CI*: 0.62 to 0.83).

#### Test-retest stability

Intra-class correlation coefficient (*ICC*) – mixed model, absolute type, average measures – was calculated for the test-retest group of 29 participants and gave a value of 0.88 (95% *CI*: 0.72 to 0.94).

Validity Table 4 shows the Spearman correlations between the GP-CORE and the other variables of the present study. The variables *diagnosed illness* and *medicate regularly* were excluded from these analyses because these two variables had very highly skewed distributions (see Table 1). For the total group, the correlations between the GP-CORE and the four background variables (gender, age, education, and cohabitant) were all not significant. However, women with a cohabitant had higher values on GP-CORE than women without a cohabitant (*r* = 0.17, *p* < .05). For men, the relation was the opposite but not significant (*r* = − 0.18, *p* > .05). The Fisher’s *z*-test indicated a significant gender difference (*z* = 2.37, *p* < .05) and was followed up by a 2 (gender) × 2 (cohabitant) ANOVA for independent measures with GP-CORE as the dependent variable. The ANOVA indicated a significant antagonistic interaction (*F*_1, 243_ = 5.48, *p* < .05, *η*_*Partial*_^2^ = 0.02) and showed that women with a cohabitant had lower well-being than men with a cohabitant but women without a cohabitant had a higher well-being than men without a cohabitant.

**Table 4:** Correlations between GP-CORE, backgrounds variables, and EQ-5D-5L at Time 1

Variables	GP-CORE			
	Total sample (n = 247)	Women (n = 184)	Men (n = 63)	Fischer’s *z* (r _Women_ – r _Men_)
Background variables				
Gender ^a^	0.06	–	–	–
Age	−0.02	− 0.01	− 0.09	0.54
Education ^b^	0.09	0.10	0.07	0.20
Cohabitant ^c^	0.09	0.17*	−0.18	2.37*
EQ-5D-5L				
Mobility	0.25**	0.21**	0.41**	−1.49
Self-care	0.26**	0.23**	0.37**	−1.04
Usual activities	0.31**	0.32**	0.30*	0.15
Pain / discomfort	0.28**	0.33**	0.11	1.56
Anxiety / depression	0.54**	0.59**	0.40**	1.71
Self-rated health	−0.38**	−0.37**	−0.44**	0.56

*Notes*. ^a^Men = 1, Women = 2; ^b^Elementary school = 1, Gymnasium or University = 2; ^c^Yes = 1, No = 2

**p < .01 (two-tailed), *p < .05 (two-tailed)

The correlations between GP-CORE and the six health-related quality of life questions were all in the expected direction and all, except one, significant. No gender differences regarding correlation between GP-CORE and the six health-related quality of life questions were observed. In summary, higher values on GP-CORE (lower psychological well-being) was associated with greater problems with mobility, greater problems connected with self-care, greater problems performing usual activities (e.g. housework, family, leisure activities), greater levels of pain/discomfort, greater levels of anxiety/depression and lower self-rated health.

### Cut-off value and sensitivity/specificity

The participants were divided based on their answers to the general health question in EQ-5D-5L. The median value of the answers for this question was calculated to be 50 points. Those respondents who rated below the median (0–49 points) were defined as having bad health (*n* = 84) and those who rated equal to or above the median (50–100 points) were defined as having good health (*n* = 163). Participants who were classified as having bad health had *M* = 1.60 (*SD* = 0.56) and participants who were classified as having good health had *M* = 1.24 (*SD* = 0.59) on the GP-CORE at Time 1 (*t*_245_ = 4.68, *p* < 0.001, Cohens *d* = 0.63). To calculate the cut-off value on the GP-CORE that could be used to separate those with bad health from those with good health, the following formula was used [26]:

1 $$\frac{M_{Bad\ health}\kern0.50em {SD}_{Good\ health}+{M}_{Good\ health}\ {SD}_{Bad\ health}}{SD_{Good\ health}+{SD}_{Bad\ health}}$$

The cut-off value was calculated to 1.43. Using this cut-off value, the sensitivity and specificity for the GP-CORE at Time 1 was estimated. The sensitivity (the percentage of those participants that rated their health as bad (0–49 points) falling over the cut-off value) was 59.5%. The specificity (the percentage of the participants that rated their health as good (50–100 points) falling on or below the cut-off value) was 62.6%.

#### Average changes

To investigate sensitivity to change for the GP-CORE, the means and bootstrapped 95% confidence intervals on GP-CORE for men and women at the three measurement occasions are shown in Table 5. A 2 (Gender) × 3 (Time) ANCOVA with cohabitant as the covariate and GP-CORE as the dependent variable was performed. The analyses showed significant time differences, *F*_2, 398_ = 3.08, *p* < .05, *η*_Partial_^2^ = 0.02, where pairwise comparisons (Bonferroni *post-hoc* tests) showed significant differences between Time 1 and Time 2 (M _Time 1_ – M _Time 2_ = 0.39, *p* < .001, Cohen’s *d* = 0.60), significant differences between Time 1 and Time 3 (M _Time 1_ – M _Time 3_ = 0.40, *p* < .001, Cohen’s *d* = 0.59) but no significant difference between Time 2 and Time 3 (M _Time 1_ – M _Time 2_ = 0.01*, p* > .05, Cohen’s *d* = 0.02). No significant gender differences were observed, *F*_1, 199_ = 3.86, *p* > .05, *η*_Partial_^2^ = 0.02. No significant interaction between gender and time was observed, *F*_2, 398_ = 0.859, *p* > .05, *η*_Partial_^2^ = 0.01. In summary, the results showed that at Time 2 well-being (as measured by GP-CORE) improved on average by 0.39 points relative to Time 1, with an associated Cohen’s *d* = 0.60 which can be interpreted as a medium effect size [27], and at Time 3 well-being was still on average 0.40 points better than at Time 1, with an associated Cohen’s *d* = 0.59 which can be interpreted as medium effect sized [27] but that there were no significant differences in well-being between Time 2 and Time 3.

**Table 5:** Arithmetic means (M) and bootstrapped 95% confidence intervals (CI) for women (*n* = 157) and men (*n* = 45) on the three measurement-occasions on GP-CORE

Gender	Time		
	Time 1	Time 2	Time 3
Women			
M (CI)	1.40 (1.29–1.48)	1.02 (0.94–1.12)	1.05 (0.96–1.15)
Men			
M (CI)	1.23 (1.08–1.41)	0.83 (0.68–0.99)	0.79 (0.64–0.96)
Total			
M (CI)	1.36 (1.28–1.43)	0.98 (0.92–1.07)	0.99 (0.91–1.07)

#### Individual changes

To further investigate the sensitivity to change of GP-CORE, it was analysed for every participant if the participant’s change between two measurement occasions is beyond what could be attributed to measurement error or in other words if it is a reliable change. The formula to calculate reliable change (see [28]) is as follows:

2 $$\pm 1.96\ {SE}_{diff}$$

where:

±1.96: gives a 95% Confidence Interval.

$${SE}_{\textrm{diff}}={SD}_1\sqrt{2}\ \sqrt{1-r}$$

*SD*_1_: standard deviation of the baseline measurement (or pre-measurement).

*r*: reliability of the measurement (e.g. Cronbach alpha coefficient) at baseline (or pre-measurement)

Changes that fall outside the interval of formula 1 are unlikely to occur more than 5% of the time by unreliability of the measurement alone and are considered as reliable changes. Using formula 1, it was analysed how many participants had reliable changes from Time 1 to Time 2, from Time 1 to Time 3, and from Time 2 to Time 3. The results are presented in Table 6. As can be seen in Table 6: after three months of intervention or homecare (at Time 2), 52 of the 227 participants (22.9%) perceived that their well-being (as measured by GP-CORE) had improved; after six months of intervention or homecare (at Time 3), 45 of the 202 participants (22.3%) perceived that their well-being had improved, and finally; the improvement between Time 2 and Time 3 is negligible, where only 7 of 202 participants (3.5%) perceived to have improved. In summary, 22.9% of the participants had better well-being after three months of intervention or homecare compared to baseline, and 22.3% of the participants had better well-being after six months of intervention or homecare compared to baseline, but the improvement plateaued and was negligible between Time 2 and Time 3.

**Table 6:** Reliable change between the three measurement occasions for the participants in the present study

	Time 1 vs Time 2 (*n* = 227)	Time 1 vs Time 3 (*n* = 202)	Time 2 vs Time 3 (*n* = 202)
Reliable deterioration	6 (2.6%)	5 (2.5%)	9 (4.5%)
No reliable change	169 (74.4%)	152 (75.2%)	186 (92.1%)
Reliable improvement	52 (22.9%)	45 (22.3%)	7 (3.5%)

## Discussion

The aim of the present study was to evaluate the psychometric properties of GP-CORE and not to evaluate the effects of the intervention per se. Because no differences were detected between the intervention group (receiving intensive reablement) and the control group (receiving standard homecare), the two groups were combined to obtain a larger group of individuals for the analyses.

It should also be noted that the GP-CORE scale has been constructed with the intention to minimize gender bias (see [14]) and that no significant differences between men and women were found in Sinclair et al.´s [15] evaluation of GP-CORE. In addition, in the present study one gender difference was found, women without a cohabitant had a higher well-being than men without a cohabitant. This interesting result is understandable if it can be assumed that traditionally men are somewhat older than their wives, that women traditionally take more responsibility than their husbands for the household (e.g., cooking and cleaning) and that the older person in the marriage is probably less healthy than the younger, and consequently usually must be taken care of by the younger person in old age [29]. Additionally, women in older age seem to experience that “things and dwelling place support and display who I am” [30]. A major part of the sample in the present study were women in single households that reported more positive well-being. This could be understood in the light of Young [30] and by referring to qualitative interview data in which twenty women of the same sample expressed having found home-ness in their social context keeping their different roles in life as a wife, a mother, a neighbor, a friend, and a colleague while in the end of life they were living alone [4]. Daily contacts with neighbors, relatives, and symbolic objects such as pictures, artwork, and furniture can establish social interactions with both alive persons and dead family members and friends.

Regarding acceptability, 87.9% (217 out of 247 participants) had answered all 14 GP-CORE questions. This is higher than observed for CORE-OM among older adults (66% for non-clinical older adults and 73% for the clinical older adults; see Barkham et al. [12]) but lower compared to GP-CORE observations among students (95.7%; see [15]). It could be noted that the three questions that had the highest non-response rate are understandably related to the age of the sample and considering them being included in the present intervention project based on their physical health limitations: “I have felt able to cope when things go wrong.`(Q4), “I have felt optimistic about my future.`(Q13), and “I have achieved the things I wanted to.`(Q14). When compared to the three questions that were answered by every participant “I have been troubled by aches, pains or other physical symptoms` (Q5), it is clear that being older is relevant to older persons receiving home care.

The reliability of the scale was satisfactory. Internal consistency (Cronbach alpha coefficient) was greater than 0.70, which by common standards can be considered satisfactory (e.g. [31]), although a bit lower than 0.87 in the developmental non-clinical student sample of GP-CORE [15]. The test-retest stability (ICC) over a period of 7–10 days showed a value equal to 0.88, which also is a satisfactory value because it exceeded 0.80 [32] and very close to what was found in the developmental sample (i.e. 0.90) [15].

The results from the present study gave support for the convergent and discriminant validity (evaluated in terms of correlations between GP-CORE and the six items of EQ-5D-5L), because the directions of the significant correlations were all as expected whereas the magnitude of the correlations did not indicate substantial overlap. The correlations between GP-CORE and the six items of EQ-5D-5L showed that higher values on GP-CORE (more distress) were associated with greater problems with mobility, greater problems connected with self-care, greater problems performing usual activities (e.g. housework, family, leisure activities), greater levels of pain/discomfort, greater levels of anxiety/depression and lower self-rated health. These obtained relations between GP-CORE and EQ-5D-5L add further evidence to convergent validity of GP-CORE, since in previous evaluations GP-CORE has been related to various other measures (see [15]). Because the CORE literature recommends that domain scores are only explored where particularly indicated clinically or for specific research interest, we did not try to produce a factor structure [16].

The cut-off value of 1.43 points in the present study is higher than the cut-off value for non-clinical older men (0.95 points) and for non-clinical older women (0.97 points) obtained for CORE-OM by Barkham et al. [12] but because of the principles from which GP-CORE items were chosen from the CORE-OM, i.e. positively keyed low intensity and no self-harm or aggression [15], a tendency to score higher in GP-CORE than in the CORE-OM seems likely. The cut off value of GP-CORE was somewhat lower than the cut-off value for clinical and non-clinical male students (1.49 points) and for clinical and non-clinical female students (1.63 points) obtained for GP-CORE by Sinclair et al. [15]. However, given the large age differences between the present sample and the student samples in Sinclar et al. [15], a difference in cut-off values does not seem unlikely. Nevertheless, given that the present cut-off value was calculated in relation to a general health measure that was split by the median, it is very preliminary and future studies should establish a cut-off value in relation to another well-being measure relevant for mental health in older adults. Unfortunately, measurement candidates validated for older adults are scarce [6]. The sensitivity (59.5%) and specificity (62.6%) of the GP-CORE instrument in the present study was much lower than sensitivity (85.7%) and specificity (83.7%) in the study by Barkham et al. [9] but could not be compared to sensitivity and specificity in the study by Sinclair et al. [15] because it was not reported. Relative to Time 1 (baseline) both at Time 2 and at Time 3 the intervention and traditional homecare improved the participants´ subjective well-being (as measured by GP-CORE) by on average 0.40 points and resulted in approximately 22% of the participants having reliably better well-being, which again gives some indication that GP-CORE is sensitive to change. This finding adds further evidence to the results from other studies on GP-CORE (done predominantly on university students) that has shown that this instrument is sensitive to changes (see [15, 33, 34]).

## Conclusions

To conclude, the GP-CORE showed satisfactory psychometric properties to be used to measure and monitor subjective well-being in older adults (> 65 years). Future studies should establish a cut-off value in relation to another well-being measure relevant for mental health in older adults.

## Data Availability

The datasets used and/or analysed during the current study available from the corresponding author on reasonable request.

## Abbreviations

CORE-OM: Clinical Outcomes in Routine Evaluation Outcome Measure
EQ-5D-5L: The EuroQol five-dimensional Health-related quality of life
GP-CORE: general population - Clinical Outcomes in Routine Evaluation
ICC: intra-class correlation coefficient
WHO: World Health Organization.
